# COVID‐19 in Latin America and the Caribbean: Two years of the pandemic

**DOI:** 10.1111/joim.13499

**Published:** 2022-04-22

**Authors:** Alvaro Schwalb, Eleonora Armyra, Melissa Méndez‐Aranda, César Ugarte‐Gil

**Affiliations:** ^1^ Instituto de Medicina Tropical Alexander von Humboldt Universidad Peruana Cayetano Heredia Lima Peru; ^2^ London School of Hygiene and Tropical Medicine London UK; ^3^ Health Innovation Lab Instituto de Medicina Tropical Alexander von Humboldt Universidad Peruana Cayetano Heredia Lima Peru; ^4^ Facultad de Ciencias y Filosofía Laboratorio de Investigación en Enfermedades Infecciosas Universidad Peruana Cayetano Heredia Lima Peru; ^5^ School of Medicine Universidad Peruana Cayetano Heredia Lima Peru

**Keywords:** SARS‐CoV‐2, COVID‐19, Latin America, vaccination, global health

## Abstract

Worldwide, nations have struggled during the coronavirus disease 2019 (COVID‐19) pandemic. However, Latin America and the Caribbean faced an unmatched catastrophic toll. As of March 2022, the region has reported approximately 15% of cases and 28% of deaths worldwide. Considering the relatively late arrival of SARS‐CoV‐2, several factors in the region were determinants of the humanitarian crisis that ensued. Pandemic unpreparedness, fragile healthcare systems, forthright inequalities, and poor governmental support facilitated the spread of the virus throughout the region. Moreover, reliance on repurposed and ineffective drugs such as hydroxychloroquine and ivermectin—to treat or prevent COVID‐19—was publicised through misinformation and created a false sense of security and poor adherence to social distancing measures. While there were hopes that herd immunity could be achieved after the region's disastrous first peak, the emergence of the Gamma, Lambda, and Mu variants made this unattainable. This review explores how Latin America and the Caribbean fared during the first 2 years of the pandemic, and how, despite all the challenges, the region became a global leader in COVID‐19 vaccination, with 63% of its population fully vaccinated.

## Graphical abstract

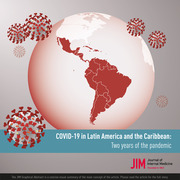

## Introduction

The coronavirus disease 2019 (COVID‐19) pandemic has not advanced similarly between countries. It has catastrophically affected some nations much more than others. Following the outbreak in Wuhan City in the Hubei Province of China in early 2020, a rapid international spread introduced the novel coronavirus, SARS‐CoV‐2, in virtually all territories around the globe [1]. In Latin America and the Caribbean (LAC), the first case was reported in Brazil on 26 February 2020, and the first death was announced shortly after in Argentina on 7 March [2, 3]. Relatively speaking, the virus was introduced late, giving the region valuable time to prepare based on strategies that were being used elsewhere. Nonetheless, its arrival was still catastrophic.

**Fig. 1:**
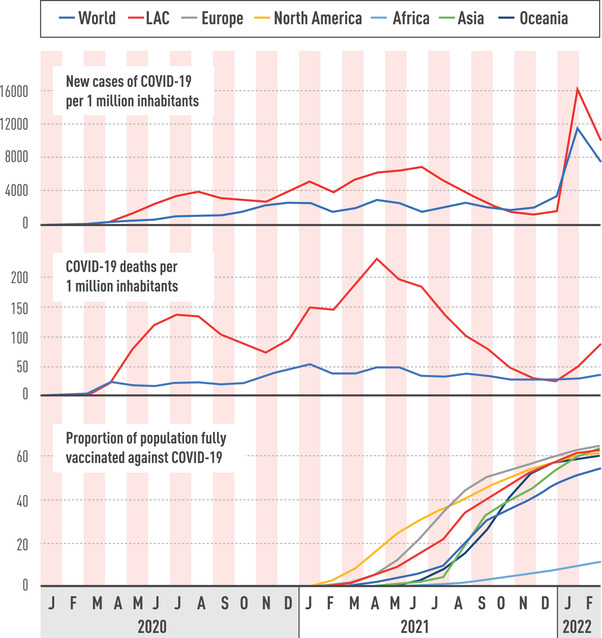
New cases, deaths, and proportion of population fully vaccinated in Latin America and the Caribbean. Source: ourworldindata.org/coronavirus.

As of 2 March 2022, 65.4 million confirmed cases and 1.65 million deaths have been reported in LAC. With a total population of more than 652 million inhabitants (8% of the world population), these amount to approximately 15% of cases and 28% of deaths reported worldwide [4, 5] (Fig. 1). Prior to the Omicron wave, LAC was the epicentre of the SARS‐CoV‐2 pandemic with the highest proportions of cases and deaths among all other regions [6]. Amidst consecutive waves, LAC has not only faced a devastating tally but also crippling social and economic costs [7]. In this review, we aim to provide an overview of how this socioeconomically and politically complex region has succumbed to the magnitude and persistence of this public health crisis [8].

## Preparedness and initial response

The Global Health Security (GHS) Index assesses the national capability to address the threat of an international infectious disease agent [9]. In the months prior to the first reported cases of COVID‐19, all LAC countries had failed to meet the high‐score band (≥66.7/100) for pandemic preparedness [9]. Brazil, Argentina, Chile, and Mexico were ranked as the most prepared LAC countries with GHS Index scores ranging from 57.6 to 59.7 [9]. This transparency in preparedness might not be easily translated into practice, with some countries overperforming during the pandemic, as in the case of New Zealand, which ranked 35th in the GHS Index report [9]. Sadly, LAC was assessed fairly, with most countries underperforming during the pandemic. In retrospect, these assessments suffer discrepancies which suggest that other factors come into play, such as the effect of exceptional leadership in times of crises. Therefore, frequent re‐evaluation of the GHS Index should be considered [10].

According to the World Health Organisation (WHO), most LAC countries fail to reach the global average of 2.9 hospital beds per 1000 inhabitants, which underlines their chronic and severe underfunding [11]. In Latin America, Argentina rises above the global average with 4.9 hospital beds per 1000 inhabitants, and both Brazil and Chile have an estimated 2.1 hospital beds per 1000 inhabitants. On the other hand, Peru and Colombia have an even lower estimate of 1.6, with Ecuador close by with 1.5 hospital beds per 1000 inhabitants [11]. For countries in the Caribbean, this estimate is even lower, with Haiti having only 0.7 hospital beds per 1000 inhabitants [11]. The same can be said about Intensive Care Unit (ICU) beds since, before the pandemic, many LAC countries had ICU bed numbers that were below the bare minimum of 6 per 100,000 inhabitants [12]. Only Brazil and Argentina reported 26 and 19 ICU beds per 100,000 inhabitants, respectively [12, 13, 14]. In the first months of the pandemic, ICUs were prioritised, and countries were able to increase their capacity slightly [12]. A massive increase was seen in Mexico, whose ICU beds rose from 2 to 25 per 100,000 inhabitants [12]. Like other countries in the region, Mexico's population displays a high prevalence of obesity, diabetes, and hypertension, which places a large proportion of them at risk of severe COVID‐19 and, consequently, ICU bed requirements [15]. Underinvestment, however, has not only been visible in hospital resources but also in the shortage of qualified healthcare workers, which is one of the biggest problems the region has to face [16]. The Pan American Health Organisation (PAHO) has estimated that around 50,000 additional physicians and nurses are required for ICU needs to be met [17]. Having both adequate staff and hospital resources is essential to meeting the healthcare demand during this critical time [18].

Considering the unpreparedness of the region, immediate actions were necessary as the introduction of SARS‐CoV‐2 loomed. Governments’ responses to the pandemic have varied greatly. However, the initial measures were very similar in regard to magnitude and timing [13, 19]. Restrictions were set in place to evaluate and, if needed, isolate travellers arriving in the last days of February 2020. Nevertheless, by the second week of March, Argentina, Colombia, Chile, and Peru had enforced total lockdowns to prevent the spread of SARS‐CoV‐2, less than 10 days after each country detected its first COVID‐19 case [19]. Additional measures, including quarantines and curfews, were also implemented [13, 19]. These restrictions were extremely effective in reducing the number of cases and, for LAC countries with precarious healthcare systems, a necessity to avoid the collapse of hospitals [20]. Nonetheless, governments eventually needed to balance the prevention of disease transmission with the repercussions of the measures in the economy.

## Socioeconomic impact and excess mortality

**Fig. 2:**
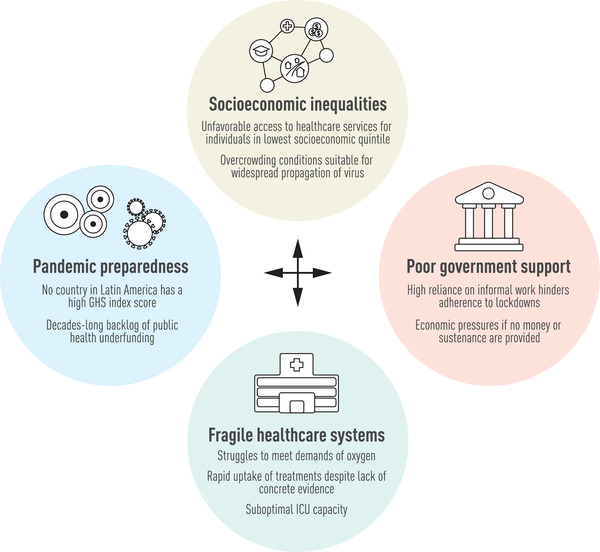
Interconnected determinants for the overwhelming death toll and crippling socioeconomic impact in Latin America and the Caribbean during the COVID‐19 pandemic.

Apart from a health crisis, COVID‐19 has also led to a humanitarian crisis in the region (Fig. 2) [21]. A decades‐long backlog of public health underfunding was a determinant of the impact encountered during the pandemic. LAC invests an average of 7.9% (range = 3.56–11.19) of the gross domestic product (GDP) while Europe and North America have a current health expenditure of 9.8% and 16.4%, respectively [22]. In January 2021, the International Monetary Fund estimated a 7.4% economic contraction in the region, with several countries projecting GDP declines of over 10% [23]. Moreover, economic recovery was expected to be lower for the LAC region in comparison to the global economic growth for both 2021 (4.1% vs. 5.5%) and 2022 (3% vs. 4.9%) [23]. Social disparities have been exacerbated by this decline.

The region as a whole reports an employment rate that is 11% lower than in pre‐pandemic times [24]. The extensive inequalities in LAC have had grave consequences for people of low socioeconomic status and those who relied on informal work for daily income [25]. Despite the early uptake of strict control measures, the existing social structure was not compatible with the governments’ pleas to stay at home and prevent transmission [13]. For some, understanding the risks involved with the community spread of SARS‐CoV‐2 led to increased willingness to follow social distancing measures [26]. Ironically, for many others, the decision to continue to work albeit exposed to a potentially deadly infection was the only way to ensure survival [27]. Although this has been the case for all countries, the strict containment policies have especially hurt the poorer regions of the world [28]. Studies suggest that household income was a determinant of the adherence to social distancing norms [26]; this was considered by most countries that opted to apply economic and social policies to support the vulnerable population [19, 29]. Measures that were not accompanied by government support in the form of money or sustenance crumbled from the economic pressures applied to households at risk [28]. For instance, urban slums were particularly struck as overcrowded conditions were suitable grounds for the widespread propagation of the virus [30, 31, 32]. Furthermore, a seroprevalence study at the peak of the first wave in Lima, Peru showed how lower socioeconomic status was linked to higher seroprevalence of SARS‐CoV‐2 infection [33]. Likewise, mortality rates were higher in deprived areas of Brazil, even in the early months of the pandemic [34]. These social inequities have been highlighted during the pandemic, as large gaps in access to healthcare have contributed to elevated out‐of‐pocket expenditure [21, 35, 36].

**Fig. 3:**
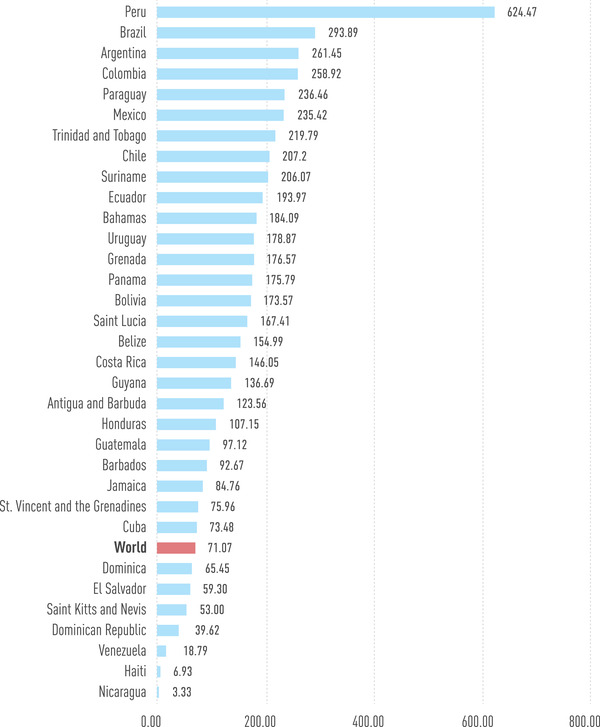
COVID‐19 mortality rates per 100,000 inhabitants in countries of Latin America and the Caribbean [Updated: 24 January 2022]. Source: worldometers.info/coronavirus/.

Despite the region's effort to mitigate the spread of the virus, the social standings ushered in a high death toll. The case‐fatality rate among LAC countries was averaged at 3.4%, with Mexico and Brazil reporting a lethality of 16.6% and 7.6%, respectively [31, 37]. Guayaquil, Ecuador's biggest city, was faced with an uncontrolled amount of deaths, where hundreds of COVID‐19 patients were left to die at home, with their bodies ending up on the streets [38]. Likewise, Ecuador's capital, Quito, experienced a gruesome sight of the pandemic as people turned to the use of cardboard coffins as a consequence of the surplus of abandoned corpses [39]. In addition, a survey revealed that seven in 10 Peruvians personally knew someone who died from COVID‐19 [40]. Nonetheless, testing capabilities were deficient in the early months, with an estimated rate of 63 tests per 100,000 inhabitants, the lowest rate worldwide [41]. Reporting deaths due to COVID‐19 proved to be a laborious task, for which many nations turned to evaluating all‐cause excess mortality as an effective alternative to track the pandemic [42]. Approximately 1.5–1.8 million excess deaths have been reported in the LAC region, with Peru currently reporting over 600 deaths per 100,000 inhabitants, the highest mortality per capita worldwide [43, 44]. For reference, the second‐highest mortality rate in the region is reported in Brazil, with over 290 deaths per 100,000 inhabitants [43]. Figure 3 provides a detailed overview of the mortality rates in the LAC countries as compared to the world average, showing how the virus has not stopped severely affecting the region throughout consecutive waves. Unfortunately, these numbers underline the deficiencies of the region's healthcare sector.

Healthcare systems vary greatly between LAC countries; however, most use a mixed system with both public and private sectors [13, 30]. While this could be seen as a means of extending access to healthcare services, it favours people in the highest income quintile compared to the lowest quintile [45]. Early measures were intended to flatten the curve and, among other reasons, ensure the continuation of the provision of healthcare. However, these fragmented and collapsed systems, with suboptimal ICU capacity, were charged with facing the pandemic head‐on [12, 13]. In most countries, hospitals were struggling to meet the demands for oxygen to treat COVID‐19 patients, which plays a vital role in patient survival. [15]. For cohorts in Peru and Mexico, hypoxemia was independently associated with elevated in‐hospital mortality of 49.6% and 30.1%, respectively [46, 47]. Brazil had to cope with providing over 340,000 cylinders (7 m^3^) of oxygen per day, with Argentina, Colombia, Mexico, and Peru trailing with approximately 100,000 cylinders each [48]. The failure to meet this necessity translated into patients’ relatives having to resort to a personal hunt for oxygen, which in many cases was met with an overwhelming amount of costs [49]. This regional oxygen shortage resulted in towering numbers of preventable deaths, for which the WHO‐led COVID‐19 Oxygen Emergency Taskforce was launched [50]. A case can be made for oxygen being as essential as electricity or water, as it is an integral resource in the management of medical conditions and its unavailability might be a death sentence for some.

The healthcare sector, however, is nothing without its staff [18]. During the last 2 years, healthcare workers have experienced insurmountable amounts of pressure and daily stressors that have severely affected their mental health [51]. This was especially the case in low‐ and middle‐income countries, where difficult triage decisions had to be made due to the deficiencies in healthcare [51]. In particular, 23.9% of healthcare professionals in LAC experienced post‐traumatic stress disorder symptoms, with Argentina and Chile scoring the highest amongst the region (26.4% and 29.8%, respectively) [52]. In addition, the fear that healthcare professionals might be carrying the virus led to them facing an enormous amount of discrimination, particularly in Colombia and Mexico [52]. Moreover, resilience has been tested constantly during the pandemic, with many falling ill. A meta‐analysis estimated that over 50% of healthcare personnel were reported to be infected with COVID‐19 just during the first 6 months of the pandemic [53]. Even worse, the WHO reported that approximately 115,500 healthcare professionals died globally as a result of COVID‐19 infection [54]. The system relies on its greatest asset, the healthcare workers. Therefore, not protecting them or overextending their reach to mend the system's deficiencies is not an option.

## “Miracle” drugs and infodemia

In the early days of the pandemic, with the alarming exponential increase of hospitalisations and deaths, the entire world yearned for a panacea. With vaccine rollout ways down the line, any medication with anecdotal success or biological plausibility (in some cases, without the latter) was repurposed and used to treat COVID‐19 patients [55]. LAC was no stranger to the global craze over the unproven effects of hydroxychloroquine, azithromycin, or ivermectin [56]. The prospect of cheap, relatively safe, and widely available medications was too good of an offer for health services to ignore. These drugs were hastily incorporated into the national management guidelines, and in parallel randomised clinical trials were started to evaluate their efficacy [57]. Over 200 intervention studies from LAC were registered in the WHO International Clinical Trials Registry Platform during the first 6 months of the pandemic. The use of hydroxychloroquine, with or without azithromycin, was the most common type of intervention evaluated in the region, with 31 studies registered in the platform [57]. However, a significant number of these trials had a small sample size (less than 200 participants) and lacked a control group. Therefore, any beneficial findings would fail to be robust and be placed under scrutiny [57]. Interest in these drugs was seen not only among researchers, but trends from Google searches in Brazil, Mexico, and Peru showed a general interest in dexamethasone, hydroxychloroquine, and, in particular, ivermectin [58].

The antiparasitic ivermectin gained attention when reports claimed that it was a potent inhibitor of SARS‐CoV‐2 replication in vitro [59]. This glimmer of hope resulted in widely publicised fame. However, toxic concentrations were needed for a potentially beneficial effect to occur [60]. In spite of this, ivermectin was quickly incorporated into management guidelines for mild and severe cases of COVID‐19 in Peru and Bolivia, before any concrete evidence of efficacy from clinical trials had been found [61]. Actually, many clinical trials studying the use of ivermectin for COVID‐19 treatment have shown no effect over placebo [62]. One of the largest trials of ivermectin was found to have evidence of both plagiarism and discrepancies in the data set [63, 64]. Before being withdrawn, the paper was cited several times and was even included in a meta‐analysis that has since been retracted [65]. Later, the drug's use extended to preventive therapy, whose ultimate effect was the creation of a false sense of security and poor adherence to proven preventive measures [66]. It got so far that shortages of ivermectin, due to high and continued demand, led to the use of veterinary formulations of the drug instead [36]. One of the most important determinants of its popularity was the fact that this medication was easily obtained over‐the‐counter and was relatively cheap [66]. Dispensation of the drug was not controlled, allowing for non‐medical recommendations from friends, neighbours, public figures on TV, advertisements, and even forwarded messages on the phone [66]. The ivermectin frenzy even reached high‐income countries where vaccination campaigns were already further along than in LAC [67].

The rapid uptake of these drugs into national guidelines despite lack of concrete evidence was not met with an equally rapid removal when there was overwhelming evidence against their use. Worst of all, it was even harder to detach these drugs from the public who continued to rely on these “miracle drugs” that they could get without restrictions at any pharmacy. A reason for this includes the overwhelming amount of news connected to treatment solutions for COVID‐19, usually originating from social media platforms. Fake news started gaining political value within the region, tempting individuals to make false claims or reach for ineffective solutions to the pandemic [68]. Trends of increased COVID‐19 mortality in LAC have been linked to higher use and trust of social media as a means of obtaining health information [69]. A tracker of infodemia—an epidemic of false or misleading information—has observed a high Infodemic Risk Index (99.1%) in Peru, the highest in the LAC region [70]. This suggests that the population is very likely to encounter a social media post with potentially harmful information about COVID‐19, with a moderate chance of re‐sharing or commenting on this information [70]. Another country in the region with a high Infodemic Risk Index was Costa Rica (72.8%), which has reported over 140 deaths per 100,000 inhabitants [43, 70]. Countries at medium risk of infodemia included Haiti, Guatemala, Colombia, and Brazil [70]. While it is understandable for the public to be willing to try anything to avoid the virus, it is more important to recognise when collective beliefs are causing harm or interfering with public health measures that do work.

## Herd immunity and variants

Early on, the herd immunity threshold was placed between 60% and 80% [71]. For some cities in LAC that were facing devastating first waves, the ill‐conceived prospect of having reached the threshold was a hopeful wish. In Manaus, Brazil, 76% of the population were found to be infected with SARS‐CoV‐2 by October 2020 [72]. Similarly, a high seroprevalence of 70% was found in Iquitos, Peru, another Amazonian city [73]. The recorded high seroprevalence of SARS‐CoV‐2 in these cities was striking compared to cities in Europe [74]. Some reports indicate the possibility of immunological cross‐reactivity with the dengue virus—which is prevalent in both Amazonian cities—explains the high seroprevalence encountered [75]. Nonetheless, they both presented a second peak, which suggests that the likelihood of herd immunity through infection might be unattainable [76]. Other studies were carried out in LAC, some of which also found high seroprevalence (Table 1). It is likely that waning immunity and/or immune evasion by new variants was responsible here [76].

**Table 1:** SARS‐CoV‐2 seroprevalence studies conducted in Latin America and the Caribbean during the COVID‐19 pandemic

Study	Country	Region/City	Date	Participants	Method	Seroprevalence (95% CI)
Figar et al. [112]	Argentina	Barrio Mugica, Buenos Aires	June 2020	Individuals aged 14 or older (*n* = 873)	ELISA antibody test	53.4% (52.8–54.1)
Rodeles et al. [113]	Argentina	Santa Fe	July–November 2020	Adults aged 18 years or older (*n* = 3000)	ELISA antibody test	8.83%
Armorim Filho et al. [114]	Brazil	Rio de Janeiro	April 2020	Blood donors without COVID‐19 contact or symptoms 30 days before donation (*n* = 2857)	Immunochromatographic assay	4.0% (3.3–4.7)
Silveira et al. [115]	Brazil	Rio Grande do Sul	April–May 2020 (three rounds of surveys)	All individuals (*n* = 13,111)	Lateral flow antibody test by finger prick blood sample	0.0048% (0.006–0.174), 0.135% (0.049–0.293), and 0.222 (0.107–0.408)
Tess et al. [116]	Brazil	Sao Paulo	May 2020	Adults 18 years or older (*n* = 463)	Chemiluminescent immunoassay by venous blood sample	6.0% (3.9–8.3)
Hallal et al. [117]	Brazil	Various sentinel cities	May and June 2020	Individuals ages 1 year or older (*n* = 25,025 and n = 31,165)	Lateral flow antibody test by finger prick blood sample	City‐level prevalence ranged from 0% to 25·4% in both surveys
De Souza Araujo et al. [118]	Brazil	Sergipe	July 2020	Individuals of all ages (*n* = 5615)	Lateral flow antibody test by finger prick blood sample	9.3% (8.5–10.1)
Pasqualotto et al. [119]	Brazil	Rio Grande do Sul	July 2020	Military police force (*n* = 1592)	ELISA antibody test	3.3%
Silva et al. [120]	Brazil	Maranhao	July–August 2020	Individuals ages 1 year or older (*n* = 3289)	Electrochemiluminescence immunoassay	40.4% (35.6–45.3)
Do Couto et al. [121]	Brazil	Sao Paulo	August 2020	Persons experiencing homelessness (*n* = 203), and shelter workers (*n* = 87)	ELISA antibody test	54.7% (47.8–61.5) in persons experiencing homelessness and 47.1% (36.6–57.6) in shelter workers
Lalwani et al. [122]	Brazil	Manaus	August–October 2020	Adults aged 18 years or older (*n* = 3046)	ELISA antibody test	29.1%
Cristelli et al. [123]	Brazil	Sao Paulo	September 2020	Kidney transplant recipients (*n* = 416)	Chemiluminescent immunoassay by venous blood sample	8.2% (5–11)
Miraglia et al. [124]	Brazil	Sao Paulo	September–December 2020	Adults 18 years or older (*n* = 272)	Immunoassay from venous blood sample	43.8% (37.7–50.0)
Cavalcante Pinto et al. [125]	Brazil	Fortaleza	November–December 2020	Individuals of all ages (*n* = 5615)	Immunochromatographic assay by finger prick blood sample	Children = 25.3%; adolescents = 29.2%; adults = 20.9%
Borges et al. [126]	Brazil	Sergipe	January 2021	Firefighters (*n* = 123)	Immunofluorescence assay by venous blood sample	45.5% (36.5–54.7)
Malagon‐Rojas et al. [127]	Colombia	Bogota	June–September 2020	Airport workers from 18 to 60 years of age (*n* = 212)	Chemiluminescent immunoassay by venous blood sample	23.58% (18.37–29.74)
Mattar et al. [128]	Colombia	Monteria	August 2020	Individuals of all ages (*n* = 1368)	ELISA antibody test	55.3% (52.5–57.8)
Colmenares‐Mejia et al. [129]	Colombia	Bucaramanga Metropolitan Area	September–December 2020	Occupational groups aged 18 years or older (*n* = 7045)	Chemiluminescent immunoassay by venous blood sample	19.5% (18.6–20.4)
Del Brutto et al. [130]	Ecuador	Atahualpa Canton	May 2020	Adults 40 years or older in rural areas (*n* = 673)	Lateral flow antibody test by finger prick blood sample	45%
Acurio‐Paez et al. [131]	Ecuador	Cuenca	August–November 2020	All individuals (*n* = 2457)	ELISA antibody test	13.2% (12–14.6)
Flamand et al. [132]	French Guiana	Various municipalities	July 2020	All individuals (*n* = 480)	ELISA antibody test	15.4% (9.3–24.4)
Muñoz‐Medina et al. [133]	Mexico	Nation‐wide	February–December 2020	All individuals without reported fever in the previous 2 weeks (*n* = 24,273)	Chemiluminescent immunoassay by venous blood sample	3.5% in February and 33.5% in December.
Diaz‐Salazar et al. [134]	Mexico	Guadalupe, Nuevo León	July 2020	Government employees (*n* = 3268)	Chemiluminescent immunoassay by venous blood sample	5.9% (5.1–6.7)
Cruz‐Arenas et al. [135]	Mexico	Mexico City	August–September 2020	Healthcare workers in a non‐COVID hospital (*n* = 299)	Immunochromatographic assay by venous blood sample	11.0%
Rodriguez‐Vidales et al. [136]	Mexico	Nuevo Leon	August–November 2020	Adults with no suggestive symptoms or prior diagnosis of COVID‐19 (*n* = 4495)	Chemiluminescent immunoassay by venous blood sample	27.1% (25.8–28.4)
Diaz‐Velez et al. [137]	Peru	Lambayeque	June–July 2020	Individuals aged 9 years or older (*n* = 2010)	Immunochromatographic assay by finger prick blood sample	29.5% (27.6–31.5)
Reyes‐Vega et al. [33]	Peru	Lima Metropolitan Area	June–July 2020	Individuals of all ages (*n* = 3212)	Immunochromatographic assay by finger prick blood sample	20.8% (17.2–23.5)
Álvarez‐Antonio et al. [73]	Peru	Iquitos	July and August 2020	Individuals of all ages (*n* = 716)	Immunochromatographic assay by finger prick blood sample	70% (67–73) at baseline and 66% (62–70) at 1 month follow‐up
Huamaní et al. [138]	Peru	Cusco	September 2020	All individuals aged 18 or older (*n* = 1924)	Chemiluminescent immunoassay by venous blood sample	38.8% (33.4–44.9)
Moreira‐Soto et al. [139]	Peru	San Martin	March 2021	All individuals aged 5 years or older (*n* = 563)	Chemiluminescent immunoassay by venous blood sample and confirmation by SARS‐CoV‐2 surrogate virus neutralisation test (sVNT)	59.0% (55–63)

The rise in new cases and the collective start of the second wave in LAC were fuelled by the emergence of the P.1 variant, officially named Gamma [77]. This lineage acquired 17 mutations of which three conferred changes in the spike protein leading to increased binding to the human ACE2 receptor [54]. Classified as a variant of concern, there was evidence of an increase in transmissibility (1.4–2.2 times) and of immune evasion conferred by past infection with non‐Gamma lineages of SARS‐CoV‐2 [77]. Using molecular clock phylogenetics, it was estimated that the Gamma variant appeared in November 2020 in Manaus, which was experiencing the lowest levels of daily new confirmed cases since the beginning of the pandemic [77]. The emergence of the Gamma variant explains the increase of cases in the region [78]. The wide propagation of this variant was also linked to a decrease in preventive measures which were dependent on the decisions of each state and municipality. A stark contrast was seen in the number of reported cases during this second wave between Amazonas and Pará, its neighbouring state [79]. Both states had presented a similar number of cases during their first waves from May to November 2020, but had then differed as cases in Amazonas soared [79]. As a result of the propagation of the Gamma variant, Brazil was reporting a high daily death toll, surpassing 300,000 confirmed total deaths from COVID‐19 [80]. At present, Brazil has one of the highest total COVID‐19 deaths, second only to the United States [43].

LAC also faced two variants of interest: Lambda (C.37 lineage) and Mu (B.1.621 lineage), with the earliest documented samples from December 2020 in Peru and January 2021 in Colombia [81]. In the case of Lambda, it quickly became the predominant variant in Peru in early March 2021 and, as with Gamma in Brazil, it fuelled a harsher second wave [82]. Beyond Peru, the variant was also found in a high proportion of cases in Chile and Argentina [82]. Similarly, in Colombia, Mu was the main determinant of the start of the third wave in March 2021, which rose above prior surges and exhibited a two‐stage peak‐within‐a‐peak [83]. Mu's transmissibility in Colombia may have been aided by the easing of restrictions to stimulate the economy [83]. The discovery of these variants should bring praise to the genomic sequencing capacity of the region, in spite of its shortcomings. Needless to say, LAC was also confronted with other variants of concern. In July 2021, Mexico had the highest number of COVID‐19 cases due to the Delta (B.1.617.2) variant [84]. Although it spread, the aforementioned “local” variants of interest were predominantly driving the new waves of 2021. At present, the Omicron (B.1.1.529) variant is sweeping through the nations, producing infection rates at all‐time pandemic highs [85]. Thankfully, an equal rise in hospitalisations or deaths is not evident.

## Vaccination

After a year of struggles and enforced social distancing measures, vaccine rollout started, albeit at a slow pace, by virtue of the COVID‐19 Vaccines Global Access (COVAX) project and negotiations with pharmaceutical companies [86]. In early March 2021, Colombia was the first country in LAC to receive a delivery of the Pfizer‐BioNTech vaccine through the COVAX program [87]. Nonetheless, it soon became apparent that distribution was not equitable. Only around 0.1% of the available vaccines worldwide were shipped to low‐income regions, which goes against COVAX's promise of fair global access to vaccines [88, 89]. Consequently, in the first semester of 2021, vaccine coverage against COVID‐19 in LAC fell short compared to coverage in Europe and North America [86]. With many countries being far from the reach of the PAHO's recommended vaccination coverage, LAC still managed to fully vaccinate (two doses) 63% of its population, the highest percentage for any region in the world to date [87]. Uruguay and Chile have vaccinated a remarkable amount of their citizens, earning their place in the top five countries worldwide [90]. LAC's vaccine triumph was a pleasant surprise considering the region's rough and painful start. Nonetheless, previous insights into barriers of vaccination suggest there is wide variability across and within countries that could lead to pockets of the unprotected population [91].

Challenges of mass vaccination have arisen and can be classified into accessibility and individual factors, including vaccine hesitancy [91]. The former poses difficulties in the delivery of mRNA vaccines, such as Pfizer/BioNTech or Moderna, whose temperature storage requirements limit their use outside of big cities [92]. For the population residing deep in the Amazon or high in the Andes, a long journey would need to be taken to reach a vaccination centre with appropriate minus 80‐degree centigrade storage, and this would need to be repeated for the second and third doses. Vaccine hesitancy, on the other hand, leads to a bigger problem, as many people are worried about the speed at which the vaccines were developed. A Chilean study in late 2020 reported that 28% of respondents were indecisive about getting vaccinated and 23% refused to get the doses overall. Of note, the questions were not referring to a specific COVID‐19 vaccine [93]. Furthermore, some LAC citizens have shown distrust in the vaccines on offer, with high scepticism over the efficacy and safety of China's Sinovac and Russia's Sputnik [94]. Fortunately, hesitancy is low compared to other regions, particularly Europe [94, 95].

Approximately 4.9 million Venezuelan migrants reside in LAC [96]. During the pandemic, they have been at higher risk of serious illness due to COVID‐19 and barriers in accessing health services [97]. Vaccination policies of most of the countries hosting the majority of Venezuelan migrants mentioned access to “migrants” in their vaccination plans. However, only Colombia and Ecuador explicitly named “Venezuelan migrants” [86, 98]. Neither Brazil nor Chile had any mention of migrants in their vaccination plans [86, 98]. Sadly, inclusion in policies did not translate into effortless access to the COVID‐19 vaccine, as migrants were required to navigate through bureaucracy and even face discrimination [89, 98]. Similarly, the indigenous populations in LAC have had struggles with vaccination, mainly due to poor access to immunisation services [99, 100]. High seroprevalence and lethality due to SARS‐CoV‐2 has been found in this vulnerable population [101]. Governments have been called to consider the difficult logistical challenges with the provision of vaccines in remote areas, like the Amazon jungle [100]. Another barrier is the lack of trust these communities have towards government health services due to prior neglect. Strengthening the relationship between them is paramount in order to narrow the gap [99].

The region learned early on that vaccination was not an immediate return to normality, even when administered at a rapid pace. Despite the fastest per‐capita COVID‐19 vaccination campaign, Chile was forced into a new strict lockdown in March 2021 amidst a soaring rise in cases [102]. While some blame was attributed to the Gamma or Lambda variant, responsibility can also be placed on the premature and sudden reopening of the country and the easing of restrictions [103]. Nevertheless, LAC can now see the positive effects that vaccines are having on the population's well‐being. Several vaccine effectiveness studies were conducted in LAC, which provided reassuring results in a time when just a fraction of the population, particularly the elderly, had received their doses [Table 2]. Now that the coverage is much greater, the effects of the vaccination programs are observable with case‐fatality among LAC countries dropping significantly. For instance, Mexico and Brazil report a lethality of 6.5% (from 16.6%) and 2.6% (from 7.6%), respectively [104]. The recent surge due to the Omicron variant is further evidence that cases decouple from hospitalisations and deaths [104]. While this might also indicate that the current wave is milder, it has the potential to cause severe disease in populations who are yet to be vaccinated or to once again overwhelm healthcare services [105].

**Table 2:** Vaccine effectiveness studies in Latin America and the Caribbean during the COVID‐19 pandemic

Study	Country	Vaccine	Doses and schedule	Participants	Effectiveness (95% CI)	Other conclusions
González et al. [140]	Argentina	Gam‐COVID‐Vac (Sputnik V)	One dose, first component	Individuals aged 60–79 years (*n* = 40,387)	78.6% (74.8–81.7) for preventing laboratory‐confirmed infections	Prevention of hospitalisation (87.6%; 95% CI: 74.8–81.7), and death (84.8%; 95% CI: 75.0–90.7)
Hitchings et al. [141]	Brazil	AZD1222 (Oxford‐AstraZeneca)	Two doses (0 and 21 days)	Adults aged 60 years or older (*n* = 137,744)	77.9% (69.2–84.2) for prevention of symptomatic SARS‐CoV‐2 infection	Prevention of hospitalisation (87.6%; 95% CI: 78.2–92.9), of ICU admission (89.9%; 95% CI: 70.9–96.5), invasive mechanical ventilation (96.5%; 95% CI 81.7–99.3), and death (93.6%; 95% CI 81.9–97.7) in the fully immunised group.
Alencar et al. [142]	Brazil	CoronaVac (Sinovac Life Sciences) and AZD1222 (Oxford‐AstraZeneca)	Two doses	Elderly people aged 75 years or older (*n* = 313,328)	Protection ratio of 132.67 (109.88–160.18) against COVID‐19‐related death with two doses.	Protection ratio of 19.31 (18.20–20.48) against COVID‐19‐related death with one dose.
Hitchings et al. [143]	Brazil	CoronaVac (Sinovac Life Sciences)	Two doses (0 and 14 days) (*n* = 53,176)	Healthcare workers, aged 18 years or older	37.1% (53.3–74.2) for prevention of symptomatic SARS‐CoV‐2 infection	At least one dose was associated with an effectiveness of 49.4% (95% CI 13.2–71.9) for prevention of symptomatic SARS‐Cov‐2 infection
Ranzani et al. [144]	Brazil	CoronaVac (Sinovac Life Sciences)	Two doses (0 and 14 days)	Individuals aged > 70 years (*n* = 43,774)	46.8% (38.7–53.8) for preventing symptomatic COVID‐19	Effectiveness against hospital admissions was 55.5% (95% CI 46.5–62.9) and against death was 61.2% (95% CI 48.9–70.5)
Jara et al. [145]	Chile	CoronaVac (Sinovac Life Sciences)	Two doses (0 and 14 days)	Participants 16 years of age or older (*n* = 10,187,720)	65.9% (65.2–66.6) for the prevention of COVID‐19 (*n* = 4,173,574)	Prevention of hospitalisation (87.5%; 95% CI: 86.7–88.2), of ICU admission (90.3%; 95% CI: 89.1–91.4) and death (86.3%; 95% CI 84.5–87.9) in the fully immunised group.
Murillo‐Zamora et al. [146]	Mexico	BNT162b2 (Pfizer‐BioNTech)	Two doses (0 and 21 days)	Healthcare workers aged 18 years and above with laboratory‐confirmed COVID‐19 (*n* = 312)	Vaccine effectiveness was 100% against severe illness after one or two doses of the vaccine	‐
Silvia‐Valencia et al. [147]	Peru	BBIPB‐CorV (Sinopharm)	Two doses (0 and 21 days)	Healthcare workers aged 18 years and above (*n* = 606,772)	50.4% (49.0–52.0) for prevention of SARS‐CoV‐2 infection after two doses	94.0% (91.0–96.0) for prevention of COVID‐19 related‐death after two doses

## Final remarks

While the battle continues, LAC is compelled to address additional problems that arose as a consequence of the pandemic. As ICU and emergency services were presented with an influx of cases, it remains to be seen how the healthcare system will fare against the population suffering disability from the long‐term effects of COVID‐19 [106]. Furthermore, children in the region have missed more school than anyone else worldwide. The effects of this disruption in education may continue to be felt for years to come [107]. Sadly, another secondary impact of the pandemic has been the distressing number of children affected by orphanhood, with an estimate of more than 1 in 1000 children in several countries in LAC [108]. Moreover, stay‐at‐home measures contributed to the rise in domestic violence and sexual abuse. Calls to help hotlines increased massively in Colombia—which received 127% more reports than usual—and in Argentina, with sexual violence rising more than two‐thirds [109]. Whilst the region has managed to show signs of economic growth, complete rebound is not expected to occur in the following years, imposing added misfortune to the citizens of LAC [110]. Despite the brutal reality and shortcomings, the pandemic has not drastically altered trust in the healthcare system [111]. The vaccination campaigns and adherence to public health guidelines have started to alleviate the pandemic, with the LAC region becoming the global leader in vaccination. The population is finally able to steadily, and safely, resume its social interactions and start down the road to recovery.

## Conflict of interest

The authors declare no conflict of interest.

## Author Contributions

Alvaro Schwalb: Investigation; Methodology; Writing – original draft; Writing – review & editing. Eleonora Armyra: Investigation; Writing – original draft; Writing – review & editing. Melissa Méndez‐Aranda: Investigation; Writing – review & editing. César Ugarte‐Gil: Methodology; Writing – original draft; Writing – final review.
